# Role of Breast Cancer Risk Estimation Models to Identify Women Eligible for Genetic Testing and Risk-Reducing Surgery

**DOI:** 10.3390/biomedicines12040714

**Published:** 2024-03-22

**Authors:** Azzurra Irelli, Leonardo Valerio Patruno, Sofia Chiatamone Ranieri, Daniela Di Giacomo, Sara Malatesta, Edoardo Alesse, Alessandra Tessitore, Katia Cannita

**Affiliations:** 1 Medical Oncology Unit, Department of Oncology, “Giuseppe Mazzini” Hospital, AUSL 04 Teramo, 64100 Teramo, Italy; leonardovalerio.patruno@aslteramo.it (L.V.P.); katia.cannita@aslteramo.it (K.C.); 2 Pathology Unit, Department of Services, AUSL 04 Teramo, 64100 Teramo, Italy; sofia.chiatamoneranieri@aslteramo.it (S.C.R.); daniela.digiacomo@aslteramo.it (D.D.G.); sara.malatesta@aslteramo.it (S.M.); 3 Department of Biotechnological and Applied Clinical Sciences, University of L’Aquila, 67100 L’Aquila, Italy; edoardo.alesse@univaq.it (E.A.); alessandra.tessitore@univaq.it (A.T.)

**Keywords:** hereditary breast and ovarian cancer syndrome, pathogenic germline variants, breast cancer risk estimation models, primary prevention, risk-reducing mastectomy, risk-reducing salpingo-ovariectomy

## Abstract

Hereditary breast and ovarian cancer (HBOC) syndrome is responsible for approximately 10% of breast cancers (BCs). The HBOC gene panel includes both high-risk genes, i.e., a four times higher risk of BC (*BRCA1*, *BRCA2*, *PALB2*, *CDH1*, *PTEN*, *STK11* and *TP53*), and moderate-risk genes, i.e., a two to four times higher risk of BC (*BARD1*, *CHEK2*, *RAD51C*, *RAD51D* and *ATM*). Pathogenic germline variants (PGVs) in HBOC genes confer an absolute risk of BC that changes according to the gene considered. We illustrate and compare different BC risk estimation models, also describing their limitations. These models allow us to identify women eligible for genetic testing and possibly to offer surgical strategies for primary prevention, i.e., risk-reducing mastectomies and salpingo-oophorectomies.

## 1. Introduction

Approximately 10% of all women with breast cancer (BC) are clinically related to hereditary breast and ovarian cancer (HBOC) syndrome [1,2,3], defined by the identification of pathogenic germline variants (PGVs) in HBOC-related genes. Although a family history of first-degree BC is significantly associated with an increased risk of BC in patients with HBOC syndrome, a suggestive family history may not be present [4].

**Figure 1:**
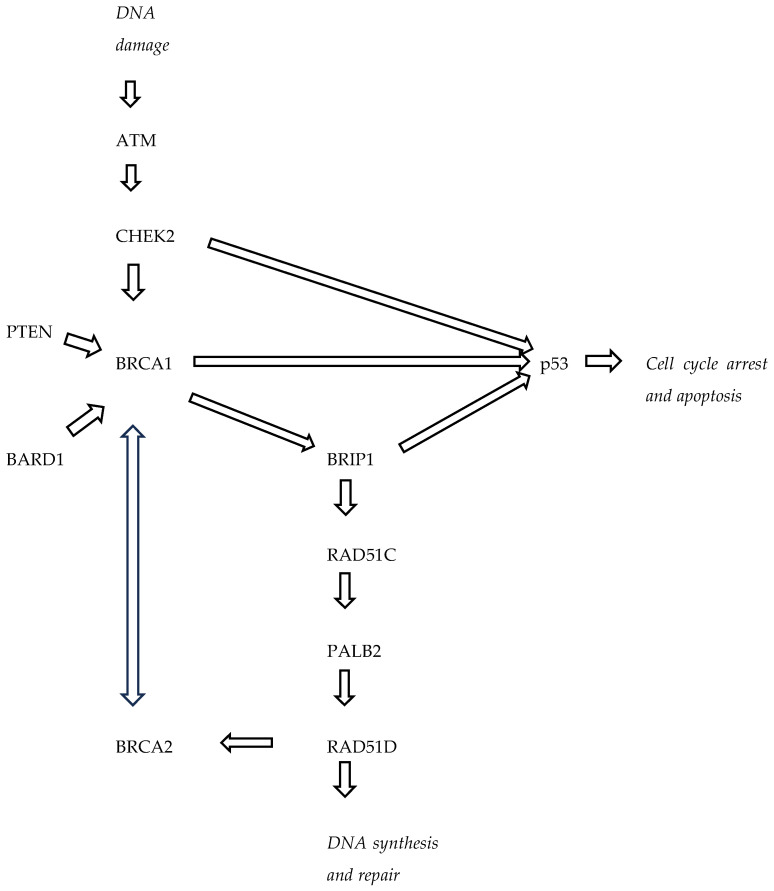
Interactions between the main proteins encoded by the HBOC-related gene.

The HBOC gene panel includes 12 genes that are both high-risk genes, i.e., a four times higher risk of BC (*BRCA1*, *BRCA2*, *PALB2*, *CDH1*, *PTEN*, *STK11* and *TP53*), and moderate-risk genes, i.e., a two to four times higher risk of BC (*BARD1*, *CHEK2*, *RAD51C*, *RAD51D* and *ATM*) (Table 1) (Figure 1) [3,5,6,7,8].

In a single-center retrospective analysis, after *BRCA1* and *BRCA2* PGVs, *CHEK2* PGVs are by far the most prevalent, followed by *ATM*, *PALB2*, and *TP53* PGVs [9]. However, it is known that the lifetime breast cancer risk for carriers of *CHECK2* PGVs is only 25–30% equal to that of *ATM* PGV carriers, but lower than that related to *PALB2* (40–60%) and *TP53* (40%) PGV carriers.

Patients with early-onset BC have the highest probability of being carriers of *BRCA1* or *BRCA2* PGVs. Conversely, patients with later-onset BC have the highest probability of being carriers of non-*BRCA1/BRCA2* PGVs [10].

In the case of a positive oncological family history, the prevalence of *BRCA1/BRCA2* PGVs is comparable to the prevalence of PGVs in HBOC-related genes not including *BRCA1/BRCA2*. On the other hand, in the absence of an oncological family history, PGVs in HBOC-related genes not including *BRCA1/BRCA2* are prevalent and can cause oncological diseases less frequently due to their low penetrance. Therefore, these women may not develop tumors during their lifetime [10].

## 2. Cancer Risks Associated with PGVs in HBOC Genes

The cancer risks associated with PGVs in HBOC genes are listed in Table 2.

Carriers of *BRCA1/BRCA2* PGV show more than a 50% risk of developing BC during their lifetime.

Carriers of *BRCA1* PGVs show: (i) an absolute risk of BC higher than 60%; (ii) an absolute risk of male BC ranging from 0.2 to 1.2%; and (iii) an absolute risk of epithelial ovarian cancer ranging from 39 to 58%. On the other hand, carriers of *BRCA2* PGVs display: (i) an absolute risk of BC higher than 60%; (ii) an absolute risk of male BC ranging from 1.8 to 7.1%; and (iii) an absolute risk of epithelial ovarian cancer ranging from 13 to 29% [5,11,12].

The cumulative risk of BC at 80 years of age is 72% for *BRCA1* carriers and 69% for *BRCA2* carriers. While the cumulative risks for *BRCA1/BRCA2* carriers through age 80 are similar, the cumulative risks up to age 50 are higher for *BRCA1* carriers.

For carriers of *BRCA1* PGV, peak incidence occurred in the 41–50 year age group, while for carriers of *BRCA2* PGV, peak incidence was in the later 51–60 year age group [13].

BRCA1-associated BCs frequently show a ductal histotype, with a negative expression of receptors for estrogen (ER) and progesterone (PR) and absence of HER2/neu amplification (“triple negative” phenotype). Instead, BRCA2-associated BCs have a luminal-like profile, i.e., positivity of ER and/or PR and absence of HER2/neu amplification, as well as a slight incidence increase of the lobular histotype [5,11].

BC patient carriers of *BRCA1/BRCA2* PGVs appear less likely to die with respect to non-carrier BC patients. This advantage may be due to the increased sensitivity of BCs with *BRCA1/BRCA2* PGVs to many chemotherapeutic agents, such as platinum-based drugs, or their increased sensitivity to immune attack [14].

The absolute lifetime risk for BC in women with *CHEK2* PGV is 25–30% higher in women with a stronger oncological family history than in those without [4,12]. Most individuals with *CHEK2* PGV develop luminal-like BCs [15].

For carriers of *ATM* PGV, risk is described as: (i) an absolute risk of BC ranging from 20 to 40% and (ii) an absolute risk of epithelial ovarian cancer ranging from 2 to 3% [4,12,15]. The missense c.7271T>G pathogenic variant of *ATM* is associated with a significant BC risk increase (three to four times) [16,17]. Most patients with *ATM* PGV develop luminal-like BCs, both HER2-negative and HER2-positive [15].

In carriers of *PALB2* PGV, risk is described as: (i) an absolute risk of BC ranging from 40 to 60%; (ii) a 10% absolute risk of contralateral BC; (iii) a 0.9% absolute risk of male BC; and (iv) an absolute risk of ovarian cancer ranging from 3 to 5%. One study showed a more than 5% lifetime risk to develop ovarian cancer [4,12].

PALB2 closely interacts with BRCA1 and BRCA2 in the homologous recombination (HR) DNA repair pathway, where the recruiting sequence at the DNA level is: BRCA1, PALB2, and then BRCA2. This suggests that PALB2 and BRCA2 may be associated to similar carcinoma risks because BRCA2 needs PALB2 to be recruited in the HR repair. Age-specific incidence of BC follows a pattern similar to that observed in *BRCA2* mutant patients, where incidence increases with age and increases steadily from age 50 onward [18].

For carriers of *TP53* PGV, an absolute risk of BC of more than 40% is estimated. *TP53* PGV appears to be associated with only about 1% of hereditary BC cases. Retrospective studies have shown that *TP53* PGVs are significantly associated with HER2-positive BC, regardless of hormone receptor status (positive or negative).

For carriers of *CDH1* PGV, an absolute risk of BC ranging from 40 to 60% is estimated. *CDH1* PGVs are primarily associated with lobular BC.

For carriers of *PTEN* PGV, an absolute risk of BC ranging from 40 to 60% and 38–50 years average age at diagnosis is described.

For carriers of *STK11* PGV, risk is reported as: (i) an absolute risk of BC ranging from 32 to 54% and (ii) an absolute risk of nonepithelial ovarian cancer (Sertoli-Leydig) from 10 to 20% [4,12].

For carriers of *BARD1* PGV, an absolute risk of mostly triple-negative BC ranging from 20 to 40% is observed [4].

For *RAD51C* and *RAD51D* PGVs carriers, it is estimated: (i) the absolute risk of BC is 10–40% and (ii) the absolute risk of epithelial ovarian cancer is 10–20%. *RAD51C* and *RAD51D* PGVs are significantly associated with triple-negative BC [4,12,19,20,21].

Besides the genes of the HBOC panel, other genes are correlated to BC.

Lynch syndrome attributed to PGVs in one of the four mismatch repair (MMR) genes (*MLH1*, *MSH2*, *MSH6*, and *PMS2*), or EPCAM gene deletionsis is also associated with an approximately 15% absolute risk of BC.

Moreover, *NF1* PGV carriers show a 20–40% absolute risk of BC, and *BRIP1* PGV a 5–15% absolute risk of developing epithelial ovarian cancer. For the latter, data to define the absolute risk of BC are, to date, not sufficient [12].

## 3. Models for Estimating BC Risk

### 3.1. Gail/BCRAT Model

The Gail model (later modified into the BC Risk Assessment Tool (BCRAT)) provides a numerical estimate of a woman’s risk of developing BC over 5 years and over her lifetime, compared to the average risk of a woman of the same age. A woman who has a 5-year relative risk ≥ 1.66 is considered worthy of preventive measures. The Gail model is based primarily on non-genetic risk factors, with limited information on family history. It takes into consideration age, age at menarche, age at first full-term birth, number of first-degree relatives diagnosed with BC, number of previous breast biopsies negative for BC, and race/ethnicity. The major limitation of the Gail model is represented by the inclusion of first-degree relatives only, and so the risk is underestimated in 50% of families with cancer in the paternal line [22,23]. The BCRAT (Figure S1) (https://bcrisktool.cancer.gov, accessed on 27 February 2024) is validated for patients aged 35 years and older in many different populations [24,25,26], but it is not very useful for women with a biopsy diagnosis of atypia, as it underestimates the overall risk [27]. The Gail model has been validated in multiple studies, undergoes periodic updates based on changes in BC incidence data, and considers competing risks of mortality other than BC. Limitations of the Gail model include: the inability to be used for individuals < 35 years old, limited use in individuals of non-European (non-white) ethnicity, only female first-degree relatives’ inclusion (paternal family history excluded), lack of inclusion of age of relatives’ BC diagnoses, family history of cancer diagnoses other than BC, prior mantle radiation therapy. In addition, it underestimates the risk of developing BC in individuals with PVGs in HBOC-related genes, those with a strong family history of BC, those with a family history of ovarian cancer in the mother or paternal family lineage, and those with atypical hyperplasia [28].

### 3.2. Claus Model

The Claus model only takes into consideration family history, i.e., history of BC in the maternal and paternal lines, first- and second-degree relatives, and age at diagnosis of BC. The major disadvantage of the Claus model is the lack of inclusion of non-hereditary risk factors. Furthermore, the Claus tables reflect the risk of the female American population in 1980, while the current risk in both the United States and Europe is higher. In conclusion, the Claus model underestimates the risk [29].

### 3.3. BRCAPro Model

The BRCAPro software package uses both the Claus and Ford models [30]. The latter is based on personal and family history of BC and ovarian cancer to identify the presence of *BRCA1/BRCA2* PGVs [31].

BRCAPro is a Bayesian computer program or statistical model for calculating an individual’s probability of being a carrier of *BRCA1/BRCA2* PGVs, based on the type of cancer and history of BC and/or ovarian cancer among relatives of the first and second degree. The BRCAPro model may also have the purpose of evaluating the risk of BC over time [32]. However, no non-hereditary risk factors are included in the model [33]. The limitations are the underestimation of the frequency of carriers in families with ovarian cancer and in families with prostate cancer, the non-applicability to ethnic minorities, the impossibility of incorporating third-degree relatives, and non-inclusion of other genes besides *BRCA1/BRCA2* [28].

### 3.4. BCSC

The BCSC Risk Calculator is an interactive tool designed to estimate a woman’s 5- and 10-year risk of developing invasive BC. Calculations of the absolute risk of BC are based on five factors: age, race/ethnicity, family history of BC in a first-degree relative (mother, sister, or daughter), history of a breast biopsy with diagnosed disease benign breast, and breast density in BI-RADS^®^ (radiological assessment of breast tissue density). Unknown values for race/ethnicity and family history are allowed. The calculator is not applicable to women who meet any of the following criteria: age younger than 35 years or older than 74 years, prior diagnosis of BC, prior diagnosis of ductal carcinoma in situ (DCIS), prior augmentation mammaplasty, prior mastectomy [34], or beyond first-degree relatives [28].

### 3.5. Tyrer-Cuzick/IBIS Model

The Tyrer-Cuzick model (also known as the IBIS risk assessment tool) was developed to evaluate the individual risk of BC over time, but also provides an estimate of the probability of finding *BRCA1/BRCA2* PGVs. This model is the first able to integrate family history with surrogate measures of exposure to oestrogen and benign breast pathology (atypical hyperplasia). In various validation processes, this model is the only one that achieved the best prediction estimates. The Tyrer-Cuzick model considers body mass index (BMI), age at menarche, parity, age at menopause (if applicable), history of benign breast pathology associated with increased risk of BC (hyperplasia, typical, LCIS), history of ovarian cancer and male BC, use of hormone replacement therapy, family history (including BC and ovarian cancer, Ashkenazi Jewish descent, and genetic testing results if performed) [35]. In the most recent version (version 8) of the IBIS risk assessment tool (http://www.ems-trials.org/riskevaluator, accessed on 27 February 2024), mammographic breast density is also included [36]. This computer model provides personalized assessment of lifelong (up to 85 years) BC risk and 10-year risk estimates. This test can be used in individuals < 35 years old and calculates the risk of *BRCA1/BRCA2* PGVs. The family history assessment includes first-, second-, and third-degree relatives (first cousins). It considers the competing risks of mortality other than BC, but does not consider the risk of mantle radiotherapy. It overestimates the risk of developing BC in Hispanic individuals, because this model has been validated primarily in white individuals in the United Kingdom, in cases of atypical hyperplasia, lobular carcinoma in situ, and dense breasts [28]. The IBIS calculator, unlike the BCRAT, can be used to “qualify” women for additional BC screening with MRI. However, this model tends to overestimate the risk for women with a biopsy diagnosis of atypia and, therefore, should not be used in this population [37].

Many studies have shown that the IBIS model, compared to the Gail and Claus models, is able to identify the highest percentage of the population at high risk [38,39,40,41].

### 3.6. BOADICEA/CanRisk Model

The “Breast and Ovarian Analysis of Disease Incidence and Carrier Estimation Algorithm” (BOADICEA) is another model for calculating the probability of *BRCA1/BRCA2* PGVs as well as the probability of BC occurrence. This model incorporates the assessment of family history of BC, ovarian cancer, prostate cancer, male BC, and pancreatic cancer with the following individual traits: sex specification; age at cancer diagnosis or age at death of all family members; genetic factors (*BRCA1*, *BRCA2*, *PALB2*, *CHEK2*, and *ATM* PGVs; polygenic risk score); height, body mass index, parity, age of first birth, age at menarche, age at menopause, use of oral contraceptive, use of hormone replacement therapy, alcohol intake; mammographic density (BI-RADS); histopathology of BC i.e., ER, PgR, HER2/neu, CK14 status, CK5/6 status; and demographic factors (country of origin, year of birth, ethnicity, such as Ashkenazi Jewish descent) [42].

The CanRisk Tool (BOADICEA v6) (https://canrisk.org, accessed on 27 February 2024) is a model for calculating BC and ovarian cancer risks based on family history and genotypes for PGVs in *BRCA1/BRCA2*, *PALB2*, *CHEK2*, *ATM*, *BARD1*, *RAD51C*, and *RAD51D* and incorporates the effects of common genetic variants (summarized as polygenic risk scores, PRS), lifestyle, hormonal and clinical characteristics, breast density, and disease histopathology. It is validated prospectively for predicting both carrier probabilities and subsequent cancer risk. It does not consider personal risk factors such as breastfeeding, previous breast biopsy and atypia, and does not include risks due to mantle radiotherapy [28]. It is the first freely accessible cancer risk prediction program to carry the European Community (EC) mark to indicate compliance with applicable safety and performance requirements for use by healthcare professionals within the European Economic Area (EEA). BOADICEA is currently recommended by several national agencies and organizations to determine eligibility for high-risk BC screening, eligibility for screening of *BRCA1/BRCA2* PGVs, and to inform BC risk management. These include the UK NICE Guidelines (https://www.nice.org.uk/guidance/cg164, accessed on 27 February 2024), the American Cancer Society, the Ontario Breast Screening Program (https://www.cancercareontario.ca/en/guidelines-advice/cancer-continuum/screening/breast-cancer-high-risk-women, accessed on 27 February 2024), and the eviQ Australian guidelines for healthcare professionals (https://www.eviq.org.au/cancer-genetics/adult/risk-management, accessed on 27 February 2024). BOADICEA’s CanRisk tool has also been incorporated into the NCCN guidelines for familial breast/ovarian cancer [43].

### 3.7. Myriad Model

The BRCA Risk Calculator (https://webapps.myriad.com/brca-risk-calculator/calc-embed.html, accessed on 27 February 2024) is based on data, periodically updated, representing characterization of deleterious PGVs by Myriad Genetic Laboratories through a clinical testing service on approximately 10,000 women. Data obtained through tests performed as part of specific research protocols are not included. Data are obtained from a routine laboratory request form, and have not been independently verified by Myriad Genetic Laboratories. The calculator asks the woman’s gender, whether the woman has Ashkenazi Jewish ancestry, whether she has been diagnosed with BC, whether anyone in the woman’s family has been diagnosed with BC under age 50, and whether someone in the woman’s family has been diagnosed with ovarian cancer [44].

The main models for estimating BC risk are shown in Table 3.

Other risk models designed to predict the probability that an individual is a carrier of the *BRCA1/BRCA2* PGVs are the PENN II model, the Lambda model, and the Couch model. The PENN II risk model (https://pennmodel2.pmacs.upenn.edu/penn2/, accessed on 27 February 2024) can be used to predict the pre-test probability that an individual has inherited *BRCA1/BRCA2* PGVs. This model does not predict BC risk. In general, individuals with at least a 5–10% chance of having a PGV in HBOC-related genes are considered good candidates for genetic testing. For the maternal and paternal sides, the model asks for the presence of Ashkenazi Jewish ancestry, the number of women in the family diagnosed with BC and synchronous ovarian cancer, the number of women in the family diagnosed with ovarian cancer or tubal cancer in the absence of BC, the number of cases in the family diagnosed with BC before the age of 50, the age of the youngest case of BC, the presence of mothers and daughters diagnosed with BC, the number of women with bilateral BC, the number of male BC cases, the presence of pancreatic cancer cases in the family, the number of prostate cancer cases in the family, and the closest relative with BC or ovarian cancer [46]. The Lambda model estimates the probability that an Ashkenazi Jewish woman is a *BRCA1/BRCA2* PGV carrier based on a point system, considering personal family history, whether she is a first-degree or second-degree relative with BC and ovarian cancer, age at diagnosis, and bilateral BC in the proband [47]. The Couch model was designed to provide probability estimates for the detection of *BRCA1* PGVs in women with a family or personal history of BC, ovarian cancer, or both [48].

## 4. Surgical Strategies for Primary Prevention of BC

In the 2000s, in the weeks following the statements of the actress Angelina Jolie, there was exponential interest in the possibility, for carriers of *BRCA1/BRCA2* PGVs, to undergo prophylactic bilateral mastectomy [49].

Prophylactic bilateral mastectomy is associated with a substantial reduction in the incidence of BC in carriers of *BRCA1 or BRCA2* PGVs [50,51,52]. Prophylactic bilateral mastectomy reduces the risk of BC in women with previous or concomitant prophylactic bilateral oophorectomy by approximately 95%, and in women with intact ovaries by approximately 90% [53].

The benefits of bilateral prophylactic mastectomy are probably greater if performed starting from the age of 30 (up to the age of 30, the cumulative risk of BC for *BRCA1/BRCA2* PGVs is only 4%); however, over 55 years of age, the evidence of benefit is weak.

To date, the data available suggest that nipple-sparing mastectomy is the preferred surgical technique compared to total mastectomy or skin-sparing mastectomy, thanks to the cosmetic outcomes, despite the possibility of leaving residual breast tissue. It follows that this technique requires continuous surveillance with gadolinium-enhanced MRI [4,54,55]. The rate of residual breast glandular tissue has been reported in up to 100% of patients and was found to be mainly associated with the type of surgeon experience [56]. However, in a study on 575 women at moderate to high risk for developing BC treated with prophylactic nipple-sparing subcutaneous mastectomy, only six women developed BC on the chest wall, with only one tumor in the nipple [54,55].

Prophylactic bilateral mastectomy was associated with lower mortality than surveillance for carriers of *BRCA1* PGVs, but for carriers of *BRCA2* PGVs, prophylactic bilateral mastectomy may lead to BC-specific survival like that of surveillance [57].

The survival benefit was observed primarily in young (<40 years) women with primary BC, featured by differentiation grade 1/2 and/or without a triple-negative phenotype, and not being treated with adjuvant chemotherapy. Contralateral risk-reducing mastectomy is associated with improved overall survival in carriers of *BRCA1/BRCA2* PGVs with a history of primary BC [58].

An increased risk of locoregional recurrence was observed in women treated with conservative therapy (HR: 4.54, *p* < 0.001). However, the risks of contralateral BC (HR: 1.51, *p* = 0.510), disease recurrence (HR: 1.16, *p* = 0.470), disease-specific recurrence (HR: 1.58, *p* = 0.200), and death (HR: 1.10, *p* = 0.660) were equivalent. Survival results after breast-conserving surgery are comparable to those of mastectomy in *BRCA* carriers. However, the risk of locoregional recurrence increases [59].

The breast-conserving surgery is an option for carriers of *BRCA1/BRCA2* PGVs who are willing to continue high-risk screening [60].

Prophylactic bilateral mastectomy is associated with frequent adverse effects, including decreased sensitivity to touch, pain, tingling, infection, oedema [61], decrease in satisfaction with body image, and sexual sensations. In seventeen case series reporting adverse events from prophylactic bilateral mastectomy with or without reconstruction, reported rates of unanticipated reoperations ranged from 4% in those without reconstruction to 64% in participants with reconstruction [62].

It is necessary to have a conversation with women regarding the degree of protection of prophylactic bilateral mastectomy, reconstruction options, risks, the residual risk of BC with age, and life expectancy, and to address the psychosocial and quality aspects of life. Although the timing of reconstruction in some patients with BC remains controversial, immediate reconstruction is appropriate for many patients undergoing prophylactic bilateral mastectomy. Occult carcinoma is found in less than 3% of women and is usually in the early stages, so postoperative therapy is rarely necessary. The benefits of an immediate rather than delayed approach to reconstruction are substantial. Thoughts on the psychological impact of the times of reconstruction have varied. It was initially thought to be advantageous for a woman to live with a mastectomy defect for several years so that she could appreciate her reconstruction more, even if the cosmetic result was not optimal. As reconstructive techniques improved, it was felt that the psychological benefit of emerging from a mastectomy with reconstruction outweighed the need for a waiting period. A study compared the preoperative psychological characteristics of women undergoing immediate versus delayed reconstruction. Those seeking immediate reconstruction had greater impairment in emotional well-being, higher levels of anxiety, and greater general mental health complaints than those opting for delayed reconstruction. This suggests that the availability of immediate reconstruction is particularly important for women’s mental health [54,55]. So, mastectomy should always be offered with immediate breast reconstruction, paying attention to the women’ psychological sphere [61,63].

Risk-reducing salpingo-oophorectomy is closely related to BC risk reduction caused by *BRCA1/BRCA2* PGVs, but the year of data publication is a critical interaction factor, and it should be noted that more recent studies have failed to find a significant reduction of the BC risk associated with risk-reducing salpingo-oophorectomy [64]. The apparent smaller effect on mortality in carriers of *BRCA2* PGVs compared to *BRCA1* PGVs may be due to the lower risk of ovarian cancer in carriers of *BRCA2* PGVs as well as the more aggressive biological characteristics of BRCA1-associated BC [55,65,66,67,68,69,70,71]. Kauff et al. demonstrated that risk-reducing salpingo-oophorectomy is associated with an approximately 85% risk reduction of BRCA1-associated gynecologic cancer and 72% risk reduction of BRCA2-associated BC. In contrast, protection from risk-reducing salpingo-oophorectomy against BRCA1-associated BC and against BRCA2-associated gynecological cancer did not reach statistical significance [72]. All studies on risk-reducing salpingo-oophorectomy and BC risk are observational in nature and subject to various forms of bias and confusion, thus limiting the conclusions that can be drawn about causality. Early studies supported a statistically significant protective association for risk-reducing salpingo-oophorectomy on the risk of BC, which is reflected in several international guidelines that recommend considering risk-reducing salpingo-oophorectomy in premenopausal women for reducing the risk of BC. However, these landmark studies have been hampered by the presence of several important biases, including informational censorship, which may have led to overestimation of any protective benefit. Contemporary studies, specifically designed to reduce some of these biases, have produced contradictory results. Taken together, there is no clear and consistent evidence for the role of premenopausal risk-reducing salpingo-oophorectomy in reducing the risk of BC in carriers of *BRCA1/BRCA2* PGVs. More recent evidence does not support a role for risk-reducing salpingo-oophorectomy to decrease BC risk for carriers of *BRCA1/BRCA2* PGVs [73].

Preventive bilateral oophorectomy was also associated with an 80% risk reduction of ovarian, fallopian tube, or peritoneal cancer in carriers of *BRCA1/BRCA2* PGVs and a 77% reduction in all-cause mortality [68,74].

After risk-reducing salpingo-oophorectomy, carriers of *BRCA1/BRCA2* PGVs show a residual risk of developing primary peritoneal carcinoma [75].

The link between *BRCA1/BRCA2* PGVs and uterine cancer is unclear. Therefore, although risk-reducing salpingo-oophorectomy is the standard treatment for women with *BRCA1/BRCA2* PGVs, the role of concomitant hysterectomy is controversial. This risk should be considered when discussing the benefits and risks of hysterectomy at the time of risk-reducing salpingo-oophorectomy in carriers of *BRCA1* PGVs [76].

Therefore, risk-reducing salpingo-oophorectomy is recommended once the desire for pregnancy is completed in women aged between 35 and 40 years with *BRCA1* PGVs, and in women aged between 40 and 45 years with *BRCA2* PGVs [4].

Risk-reducing salpingectomy with delayed oophorectomy has gained interest for women at high risk for tubo-ovarian cancer, as there is compelling evidence that especially high-grade serous carcinoma originates in the fallopian tubes [77], but it is not recommended outside of a clinical trial setting [4].

There are no data on the benefit of bilateral prophylactic mastectomy for carriers of *CHEK2*, *ATM*, *TP53*, *NBN*, *PTEN*, *STK11*, *BARD1*, *MSH6*, and *PMS2* PGVs, but this procedure can be considered on a case-by-case basis based on family history. Prophylactic bilateral mastectomy can be considered for carriers of *PALB2* PGVs, while it can be discussed in cases of *CDH1* PGVs. Risk-reducing salpingo-oophorectomy can be considered in women who have completed pregnancy and who are carriers of *PALB2* PGVs at an age > 45 years, of *RAD51C* and *RAD51D* PGVs at the age of 45–50 years, of *NF1* PGVs at an age > 45 years, and *BRIP1* PGVs from 45 to 50 years of age [12]. *CHEK2*, *NBN*, *PTEN*, *MSH6*, and *PMS2* PGVs are not associated with the risk of ovarian cancer; therefore, bilateral prophylactic salpingectomy is not indicated [12,77,78,79,80].

Large studies in women with ovarian cancer have shown that there can be a slightly increased risk of ovarian cancer in carriers of *ATM* PGVs, but there is currently insufficient evidence to recommend risk-reducing salpingo-oophorectomy.

## 5. Conclusions

Up to 20% of BCs arise in carriers of HBOC-related PGVs. The early identification of healthy carriers allows clinicians to activate primary prevention measures that can significantly reduce the incidence and/or mortality from BC. In addition to individuals at hereditary risk on a monogenic basis, there are individuals at increased risk on a multifactorial family basis. For the most common neoplasms, in fact, sharing both constitutional and exogenous risk factors with affected family members can determine significantly higher risks compared to the general population. Screening programs designed for the standard risk population may be insufficient due to starting age, frequency, and type of tests for early diagnosis in carriers at increased risk; therefore, it appears necessary to pursue the personalization of preventive actions by identifying those at high risk and by setting up intensified surveillance and specific prevention programs that complement screening. In Italy, the National Prevention Plan 2020–2025 promotes the adoption of organized pathways for the prevention of BC (and ovarian cancer) associated with *BRCA1/BRCA2* PGVs, with the activation of a Diagnostic-Therapeutic-Assistance Path called “High Hereditary-Familial Risk for people carrying BRCA pathogenetic variants” [81,82].

Annual mammography screening appointments could represent an ideal opportunity to administer screening tools to improve referrals to genetic counseling. Breast imaging centers could, therefore, serve as strategic locations for identifying women at increased risk of BC based on family cancer history who would benefit from genetic counseling and genetic testing [2].

Women should be enrolled in regular screening programs involving the administration of questionnaires according to, for example, the Tyrer-Cuzick model, to identify those at highest risk to be referred for onco-genetic counseling and possible genetic testing. This strategy could be feasible and effective, trying to guarantee its application to the entire population of women undergoing screening.

## Figures and Tables

**Table 1:** Lifetime cancer risk in BC for HBOC.

Gene	Lifetime Cancer Risk in BC
*BRCA1*	>60%
*BRCA2*	>60%
*PALB2*	40–60%
*TP53*	40%
*CDH1*	40%
*PTEN*	40%
*STK11*	40%
*CHEK2*	25–30%
*ATM*	25–30%
*BARD1*	20%
*RAD51C*	20%
*RAD51D*	10%

**Table 2:** Cancer risks associated with PGVs in HBOC genes.

	Absolute Risk of BC	Absolute Risk of Male BC	Absolute Risk of Epithelial Ovarian Cancer	Absolute Risk of Non-Epithelial Ovarian Cancer (Sertoli-Leydeg)	Molecular Profile/Histotype
*BRCA1*	>60%	0.2–1.2%	39–58%	-	Triple-negative
*BRCA2*	>60%	1.8–7.1%	13–29%	-	Luminal-like
*CHEK2*	25–30%	-	-	-	Luminal-like
*ATM*	20–40%	-	2–3%	-	-
*PALB2*	40–60%	0.9%	3–5%	-	-
*TP53*	>40%	-	-	-	HER2-positive
*CDH1*	40–60%	-	-	-	Lobular
*PTEN*	40–60%	-	-	-	-
*STK11*	32–54%	-	-	10–20%	-
*BARD1*	20–40%	-	-	-	-
*RAD51C/RAD51D*	10–40%	-	10–20%	-	Triple-negative
*MMR* genes	<15%	-	-	-	-
*NF1*	20–40%	-	-	-	-
*BRIP1*	-	-	5–15%	-	-

**Table 3:** Models for estimating BC risk [45].

Model	Gail/BCRAT	Claus	BRCAPro	Tyrer-Cuzick/ IBIS	BCSC	Boadicea/CanRisk	Myriad
Outcome predicted	Invasive BC	Invasive BC/DCIS	Invasive BC	Invasive BC	Invasive BC	Invasive BC/DCIS + ovarian cancer + risk of *BRCA1/BRCA2*, *PALB2*, *CHEK2*; *ATM*, *RAD51D*, *RAD51C*, *BARD1* and *BRIP1* PGVs	Invasive BC/DCIS + ovarian cancer + risk of *BRCA1/BRCA2* PGV
Age, range	35–90	20–79	Any	19–85	35–74	Any	6–97
Age at menarche	X			X		X	
Age at first birth	X			X		X	
Age at menopause				X		X	
Replacement hormone therapy				X		X	
BMI				X			
Race/ethnicity	X		X	X	X		
Jews			X	X			X
Prior biopsy	X			X	X		
Atypical hyperplasia	X			X	X		
LCIS	X			X	X		
Breast density				X	X	X	
Family members (grade)	I	I/II	Any	I/II/III	I		I/II
Age at diagnosis		X	X	X			X
Bilateral breast cancer			X	X			X
Ovarian cancer			X	X			X
Male breast cancer			X				X

## Supplementary Materials

The following supporting information can be downloaded at: https://www.mdpi.com/article/10.3390/biomedicines12040714/s1, Figure S1: Flow chart of The Breast Cancer Risk Assessment Tool (BCRAT), also known as The Gail Model.
