# Supplementary material for: Role of Breast Cancer Risk Estimation Models to Identify Women Eligible for Genetic Testing and Risk-Reducing Surgery
Source: Biomedicines. 2024 Mar 22;12(4):714. doi: 10.3390/biomedicines12040714 (PMC11048717; doi:10.3390/biomedicines12040714)
Supplement: Supplementary file 1 [file biomedicines-12-00714-s001.zip › biomedicines-2914767-SI.pdf]

Figure S1. Flow chart of The Breast Cancer Risk Assessment Tool (BCRAT), also known as The Gail Model.

1. Does the woman have a medical history of any breast cancer or of ductal carcinoma in situ (DCIS) or lobular carcinoma in situ (LCIS) or has she received previous radiation therapy to the chest for treatment of Hodgkin lymphoma? (When you answer "yes" to this question, you are not eligible to use this tool.)
   - Yes
   - No

2. Does the woman have a mutation in either the BRCA1 or BRCA2 gene, or a diagnosis of a genetic syndrome that may be associated with elevated risk of breast cancer? (When you answer "yes" to this question, you are not eligible to use this tool.)
   - Yes
   - No
   - Unknown

3. What is the patient's age?
   - Select age
     - range: 35-85

4. What is the patient's race/ethnicity?
   - Select
     - White
     - African American
     - Hispana/Latina
     - Asian American
     - American Indian or Alaskan Native
     - Unknown

5. What is the sub race/ethnicity or place of birth?
   - Select
     - Chinese
     - Filipino
     - Hawaiian
     - Pacific Islander
     - Japanese
     - Other Asian
     - Born outside the US
     - US born

6. Has the patient ever had a breast biopsy with a benign (not cancer) diagnosis?
   - Yes
   - No
   - Unknown

7. How many breast biopsies with a benign diagnosis has the patient had?
   - 1

- 2 or more

8. Has the patient ever had a breast biopsy with atypical hyperplasia?
    - Yes
    - No
    - Unknown

9. What was the woman's age at the time of her first menstrual period?
    - 7 to 11
    - 12 to 13
    - 14 or older

10. What was the woman's age when she gave birth to her first child?
    - Select
        - No births
        - <20
        - 20-24
        - 25-29
        - 30 or older
        - Unknown

11. How many of the woman's first-degree relatives (mother, sisters, daughters) have had breast cancer?
    - None
    - One
    - More than one
    - Unknown
